# Clinical Outcome of TAVI vs. SAVR in Patients with Severe Aortic Stenosis

**DOI:** 10.3390/jcm12165236

**Published:** 2023-08-11

**Authors:** Chayakrit Krittanawong, Hafeez Ul Hassan Virk, Joshua Hahn, Zhen Wang, Fu’ad Al-Azzam, Mahboob Alam, Samin Sharma, Hani Jneid

**Affiliations:** 1 Cardiology Division, New York University Langone Health and New York University School of Medicine, New York, NY 10016, USA; 2 Harrington Heart & Vascular Institute, University Hospitals Cleveland Medical Center, Case Western Reserve University, Cleveland, OH 44106, USA; 3 Division of Cardiology, University of Texas Medical Branch, Houston, TX 77059, USA; 4 Robert D. and Patricia E. Kern Center for the Science of Health Care Delivery, Mayo Clinic, Rochester, MN 55905, USA; 5 Division of Health Care Policy and Research, Department of Health Sciences Research, Mayo Clinic, Rochester, MN 55905, USA; 6 Division of Cardiovascular Surgery, Mayo Clinic, Rochester, MN 55905, USA; 7 The Texas Heart Institute, Baylor College of Medicine, Houston, TX 77030, USA; 8 Cardiac Catheterization Laboratory of the Cardiovascular Institute, Mount Sinai Hospital, New York, NY 10029, USA

## 1. Introduction

The utilization of transcatheter aortic valve implantation (TAVI) has become the treatment of choice in patients with severe aortic stenosis (AS) with intermediate-to-high surgical risk for surgical aortic valve replacement (SAVR). Although TAVI is currently proven to be safe and effective in such patients, limited head-to-head data exist regarding the safety and efficacy of TAVI versus SAVR with regard to long-term clinical endpoints in select patient populations. Thus, we assessed the safety and efficacy of surgical versus transcatheter interventions on both short- and long-term clinical outcomes in patients with severe AS.

## 2. Methods

We systematically performed a bioinformatic search of Ovid MEDLINE, Ovid Embase, Ovid Cochrane Database of Systematic Reviews, Scopus, and Web of Science from database inception in 1966 to January 2023 for all original studies (randomized controlled trials and observational studies) that evaluated patients with severe AS who underwent either TAVI or SAVR. Study groups were defined by allocation to TAVI or SAVR. The primary endpoints evaluated were all-cause mortality and 30-day mortality. Secondary clinical endpoints included stroke, new-onset AF, the need for a permanent pacemaker, vascular complications, respiratory complications, cardiac tamponade, and the need for a blood transfusion. Two clinicians extracted and analyzed the data using a standard extraction form that was then reviewed by other clinicians. Discrepancies were resolved through consensus deliberation. The Hartung–Knapp–Sidik–Jonkman (HKSJ) random-effects model was used to pool risk ratios (RRs) and corresponding 95% confidence intervals (CIs) were calculated. Heterogeneity across trials was estimated with I^2^ statistics (I^2^ < 25% suggesting low heterogeneity).

## 3. Results

**Figure 1:**
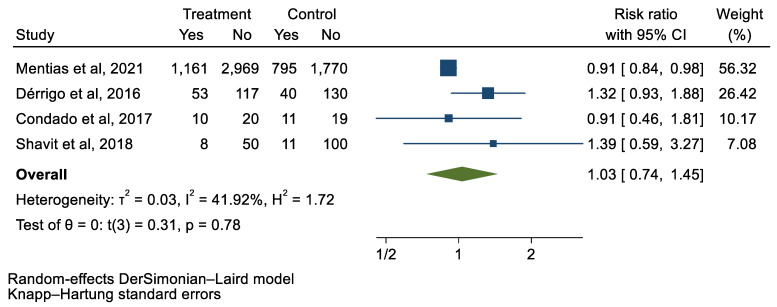
Forest plot and pooled risk ratio between TAVI and SAVR and all-cause mortality.

**Figure 2:**
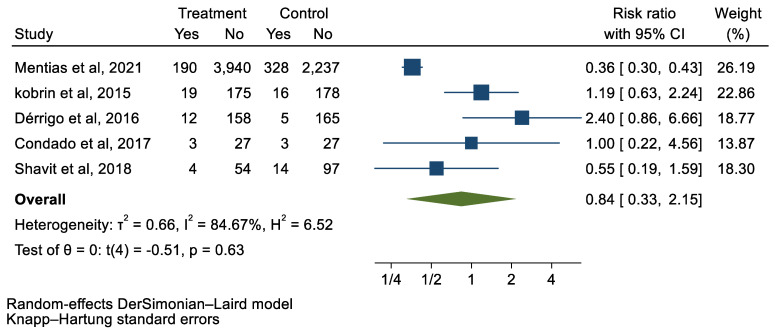
Forest plot and pooled risk ratio between TAVI and SAVR and 30-day mortality.

**Figure 3:**
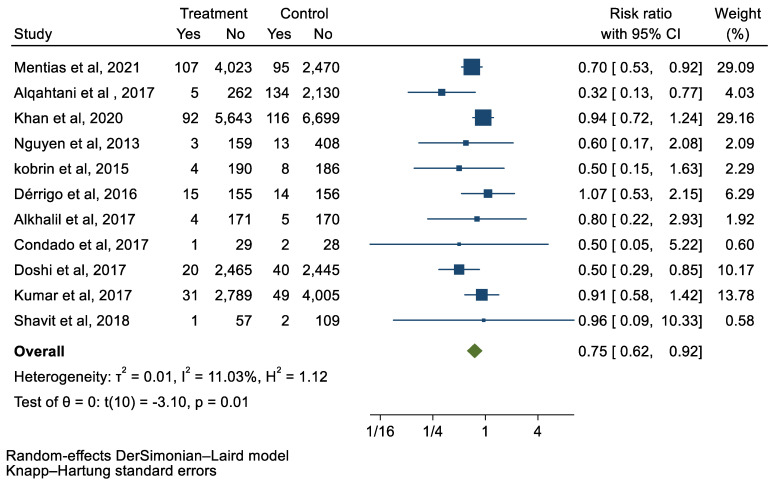
Forest plot and pooled risk ratio between TAVI and SAVR and stroke.

**Figure 4:**
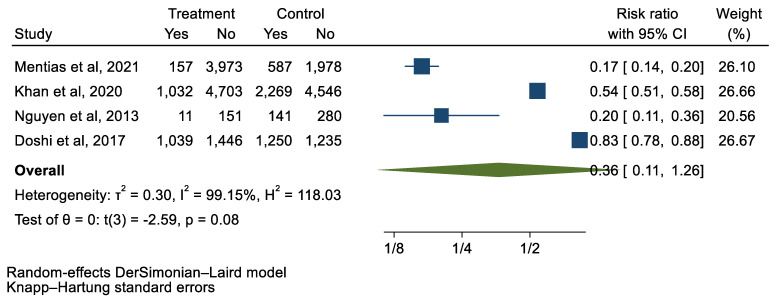
Forest plot and pooled risk ratio between TAVI and SAVR and new-onset AF.

**Figure 5:**
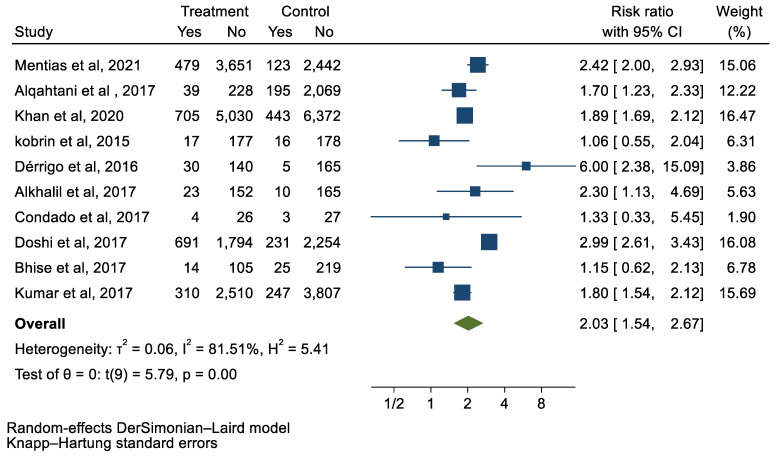
Forest plot and pooled risk ratio between TAVI and SAVR and PPM.

**Figure 6:**
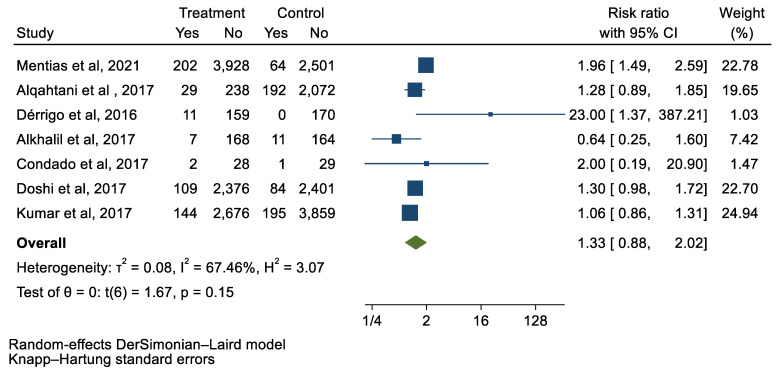
Forest plot and pooled risk ratio between TAVI and SAVR and vascular complications.

**Figure 7:**
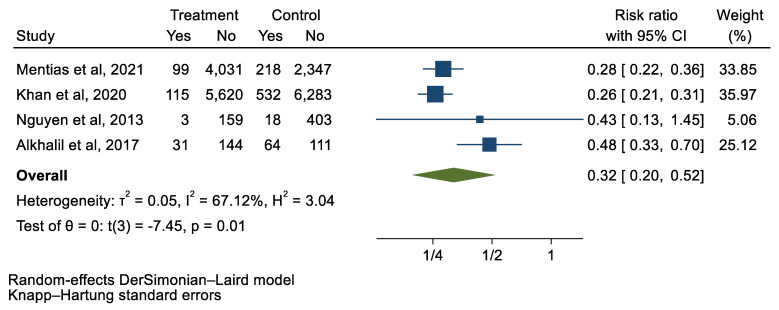
Forest plot and pooled risk ratio between TAVI and SAVR and respiratory complications.

**Figure 8:**
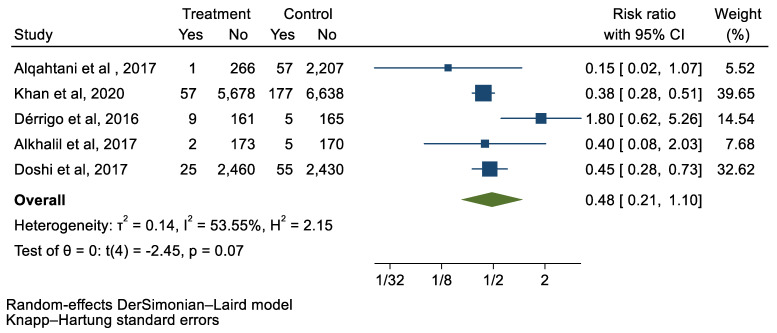
Forest plot and pooled risk ratio between TAVI and SAVR and cardiac tamponade.

**Figure 9:**
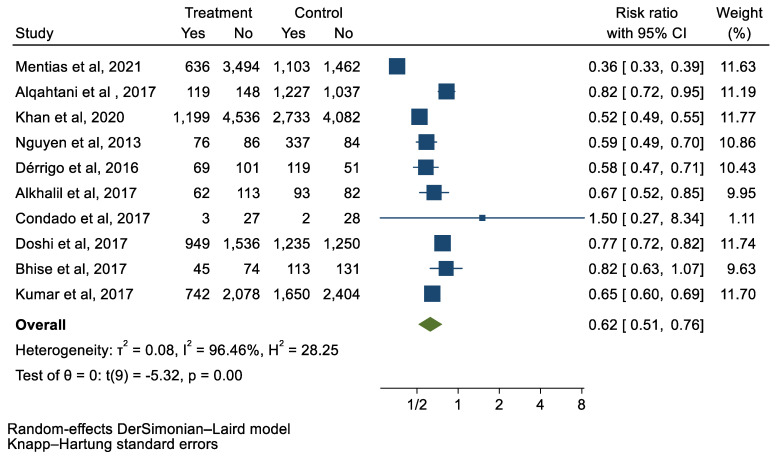
Forest plot and pooled risk ratio between TAVI and SAVR and the need for blood transfusion.

We analyzed 12 studies representing 37,825 patients who underwent either TAVI or SAVR. The evaluated endpoints were 30-day mortality, death, stroke, new-onset atrial fibrillation, the need for permanent pacemaker placement, vascular complications, respiratory complications, and cardiac tamponade. The average age of patients was 68.9 years, with 64.4% of the patient population categorized as male. There was no statistically significant difference between TAVI and SAVR in regard to the endpoints of death (a risk ratio of 1.03 with a 95% CI of 0.74–1.45), new-onset atrial fibrillation (a risk ratio of 0.36 with a 95% CI of 0.11–1.26), vascular complications (a risk ratio of 1.33 with a 95% CI of 0.88–2.02), 30-day mortality (a risk ratio of 0.84 with a 95% CI of 0.33–2.15), and cardiac tamponade (a risk ratio of 0.48 with a 95% CI 0.21–1.10). TAVI was associated with a decreased risk of stroke (a risk ratio of 0.75 with a 95% CI of 0.62–0.92) and a decreased risk of respiratory complications (a risk ratio of 0.32 with a 95% CI of 0.2–0.52). However, TAVI was associated with an increased risk of requiring a permanent pacemaker (a risk ratio of 2.03 with a 95% CI of 1.54–2.67). Figure 1, Figure 2, Figure 3, Figure 4, Figure 5, Figure 6, Figure 7, Figure 8 and Figure 9 demonstrate the results of the meta-analyses.

## 4. Discussion

In the current meta-analysis, we observed that TAVI was at least noninferior as compared to SAVR in regard to the primary endpoints of 30-day mortality and all-cause mortality. Additionally, patients undergoing TAVI had no statistically significant differences in all secondary endpoints evaluated, with the exception of the need for permanent pacemaker placement. These findings are similar to prior head-to-head randomized clinical trials demonstrating that TAVI has comparable clinical outcomes to SAVR across a wide spectrum of surgical risks [1]. However, the available follow-up time periods varied and there remains a paucity of long-term outcome data. In a meta-analysis of randomized clinical trials by Siontis et al., TAVI was associated with a reduction in all-cause mortality and stroke at 2 years, irrespective of baseline surgical risk [2]. Furthermore, recently presented trial data in exclusively low-surgical-risk patients demonstrated safety and efficacy outcomes were noninferior in patients undergoing TAVI when compared to SAVR at 3 years [3].

TAVI first emerged as a legitimate treatment option for severe AS in 2012 when the 2012 ACCF/AATS/SCAI/STS Expert Consensus Document on Transcatheter Aortic Valve Replacement was published. This document essentially maintained that TAVI could be considered as an appropriate therapeutic strategy for patients at prohibitive surgical risk for SAVR. This consensus did not give additional recommendations for TAVI, as there was a paucity of data on TAVI at the time [4]. These initial consensus statements were provided in light of the initial landmark PARTNER trial in 2010. In the initial PARTNER trial, patients who were not deemed surgical candidates were assigned to either standard therapy (balloon aortic valvuloplasty) or TAVI with a bovine pericardial valve. The study found that TAVI reduced the rates of death from any cause and decreased cardiac symptoms, but did cause an increase in stroke and vascular complications when compared with the standard surgical therapy [5]. Multiple subsequent randomized clinical trials have demonstrated the robust safety and efficacy of TAVI regardless of surgical risk. These data have contributed to the most recent guidelines for TAVI that come from the 2020 ACC/AHA Guidelines for the Management of Valvular Heart Disease. The authors recommend that deciding to treat severe aortic stenosis with either SAVR or TAVI needs a consideration of patient factors, procedural factors, and prosthesis factors. Specifically, there needs to be a multidisciplinary heart team involved in this process. Notwithstanding, both the current ACC/AHA and ESC guidelines give a class-I recommendation for TAVI in certain populations such as elderly (>80 years old) patients of any surgical risk category with a <10-year life expectancy, and in elderly patients at high or prohibitive risk for mortality from SAVR with a life expectancy of ≥1 year. SAVR is still recommended in younger patients (<65 years old) with a life expectancy >20 years; in asymptomatic patients with severe AS who have other indications for intervention (e.g., abnormal exercise test, very severe AS, rapid progression, and elevated brain natriuretic peptide); and in those whose anatomy, especially iliofemoral vascular access, is unsuitable for TAVI. The guidelines acknowledge certain limitations, such as how data for the TAVI valves were only followed for 5 years after implantation, and more data are needed for longer periods to expand to current recommendations. As TAVI gains more utility, future studies are needed to address the need for, and outcomes of operation after, the procedure [6].

In the current meta-analysis, we demonstrated that TAVI was noninferior to SAVR in regard to the endpoints of death, stroke, vascular complications, respiratory complications, 30-day mortality, new-onset atrial fibrillation, and tamponade. Given these findings, it could be argued that there could be more of a role for TAVI than just specifically for the patient population mentioned in the 2020 ACC/AHA Guidelines. As more research is conducted over longer periods of time, the guidelines may need to be updated to reflect this change. It is important to list the limitations of our meta-analysis. Every study that was part of our meta-analysis differed in its design, definitions, demographics of patients, comorbidities, and definition of outcomes.

In conclusion, this meta-analysis supports the use of TAVI in the treatment of severe aortic stenosis as it is noninferior as compared to SAVR in regard to the endpoints of death, stroke, vascular complications, respiratory complications, 30-day mortality, new-onset atrial fibrillation, and tamponade. Further studies need to be conducted that follow patients who have undergone TAVI for at least 10 years and assess for any complications or new/repeat procedures. Having these data will, thus, impact future guidelines and may offer a role of TAVI to more patients.
